# Habit Reversal Therapy: An Underutilized Treatment for Atopic Dermatitis

**DOI:** 10.7759/cureus.74095

**Published:** 2024-11-20

**Authors:** Jason R Kreutz, Fatemeh Jafarian

**Affiliations:** 1 Department of Medicine, University of Calgary, Calgary, CAN

**Keywords:** atopic dermatitis, cbt, distraction techniques, eczema, habit reversal therapy

## Abstract

Atopic dermatitis (AD) is a common inflammatory skin condition characterized by a history of recurring pruritic lesions that are worsened by scratching. Therapeutic outcomes may be optimized by minimizing the scratching of pruritic AD lesions, which is often particularly challenging for pediatric populations. Alongside topical and systemic therapies, research supports the use of habit reversal therapy for AD to mitigate the urge to scratch. North American guidelines on the management of AD should unequivocally endorse the use of habit reversal therapy. This would align with guidelines in the EU and the UK as well as the emerging body of evidence in the literature.

## Editorial

Atopic dermatitis (AD), often referred to as eczema, is a common inflammatory skin condition occurring in both children and adults. It typically involves dry, red, scaly skin lesions that are itchy and can sometimes ooze. Itching is the most common symptom arising from AD. It is essential to avoid scratching itchy AD lesions, as this is known to exacerbate illness symptoms, prolong treatment, and reduce quality of life [1]. Moisturizers, topical steroids, calcineurin inhibitors, PDE4 inhibitors, monoclonal antibodies, and JAK inhibitors are widely accepted as effective therapeutic interventions for AD [1]. Research has shown that implementing concurrent psychodermatological techniques to reduce itching can further improve treatment outcomes [2].

One such intervention for AD is habit reversal therapy, which is a form of cognitive behavioral therapy [2]. Habit reversal training is effective for managing AD by targeting the itch-scratch cycle. The efficacy of habit reversal in treating AD in both kids and adults has been supported in the literature, including an RCT examining reductions in disease severity and quality of life outcomes, although further RCTs may be useful [2]. Examples of researched distraction techniques to prevent itching in both adults and children with AD include deep breathing, guided relaxation exercises, and desensitization methods amongst other techniques. For children in particular, using toys, books, or videos that the child enjoys may draw their attention away from the desire to itch. Habit reversal techniques for AD like the aforementioned may be disseminated to patients and parents through tailored resources or counselling during visits with healthcare professionals.

In the opinion of the authors, simple distraction methods to reduce itching in AD are underutilized in the North American context. The American Academy of Dermatology originally released clinical guidelines for AD in 2014, which have been subsequently updated [1]. Habit reversal therapy was briefly referenced alongside other psychological interventions (i.e., autogenic training, biofeedback, CBT, etc.) but it was not explicitly recommended as an effective adjunct for breaking the itch-scratch cycle. In contrast, the British Association of Dermatologists lists habit reversal therapy as a beneficial adjunct for many patients with AD [3]. The European guidelines also note that habit reversal therapy has positive effects on AD management [4].

In a 2016 letter to the British Journal of Dermatology, Daunton et al. stated that the need for additional AD research should not impede the thoughtful implementation of habit reversal therapy in appropriate clinical contexts [5]. This statement continues to be relevant nearly a decade later, particularly in the North American context. Not only has most of the original data on habit reversal therapy in AD come from Europe, but the merits of habit reversal therapy and other psychological interventions deserve greater emphasis in North American guidelines. The implementation of cost-effective interventions like habit reversal therapy for AD in combination with effective topical or systemic therapies continues to hold untapped potential to improve patient outcomes. North American guidelines on AD management ought to recommend habit reversal therapy in keeping with their European counterparts.
